# A Novel Method of Outcome Assessment in Breast Reconstruction Surgery: Comparison of Autologous and Alloplastic Techniques Using Three-Dimensional Surface Imaging

**DOI:** 10.1007/s00266-020-01749-4

**Published:** 2020-05-13

**Authors:** Robin Hartmann, Maximilian Weiherer, Daniel Schiltz, Stephan Seitz, Luisa Lotter, Alexandra Anker, Christoph Palm, Lukas Prantl, Vanessa Brébant

**Affiliations:** 1 University Center of Plastic, Aesthetic, Hand and Reconstructive Surgery, University Hospital Regensburg, Franz-Josef-Strauß-Allee 11, 93053 Regensburg, Germany; 2 Regensburg Medical Image Computing (ReMIC), Ostbayerische Technische Hochschule Regensburg (OTH Regensburg), Regensburg, Germany; 3 Department of Obstetrics and Gynecology, Caritas Hospital St. Josef, University of Regensburg, Regensburg, Germany; 4 Regensburg Center of Biomedical Engineering (RCBE), OTH Regensburg and Regensburg University, Regensburg, Germany

**Keywords:** Three-dimensional imaging, Breast reconstruction, Breast symmetry, Digital anthropometry

## Abstract

**Background:**

Breast reconstruction is an important coping tool for patients undergoing a mastectomy. There are numerous surgical techniques in breast reconstruction surgery (BRS). Regardless of the technique used, creating a symmetric outcome is crucial for patients and plastic surgeons. Three-dimensional surface imaging enables surgeons and patients to assess the outcome’s symmetry in BRS. To discriminate between autologous and alloplastic techniques, we analyzed both techniques using objective optical computerized symmetry analysis. Software was developed that enables clinicians to assess optical breast symmetry using three-dimensional surface imaging.

**Methods:**

Twenty-seven patients who had undergone autologous (*n* = 12) or alloplastic (*n* = 15) BRS received three-dimensional surface imaging. Anthropomorphic data were collected digitally using semiautomatic measurements and automatic measurements. Automatic measurements were taken using the newly developed software. To quantify symmetry, a Symmetry Index is proposed.

**Results:**

Statistical analysis revealed that there is no difference in the outcome symmetry between the two groups (*t* test for independent samples; *p* = 0.48, two-tailed).

**Conclusion:**

This study’s findings provide a foundation for qualitative symmetry assessment in BRS using automatized digital anthropometry. In the present trial, no difference in the outcomes’ optical symmetry was detected between autologous and alloplastic approaches. Level of evidence Level IV.

**Level of Evidence IV:**

This journal requires that authors assign a level of evidence to each article. For a full description of these Evidence-Based Medicine ratings, please refer to the Table of Contents or the online Instructions to Authors www.springer.com/00266.

## Introduction

Breast cancer remains the most common malignant neoplasm in women [1]. In 2020, an estimated 77 600 new diagnoses are expected in Germany [2]. Almost thirty percent of newly diagnosed patients require a mastectomy, and approximately a third of these patients receive breast reconstruction surgery (BRS) [3].

Given this scenario, BRS has become an indispensable tool for patients undergoing a mastectomy [4]. In BRS, securing symmetry is a primary goal for both patients and plastic surgeons [5].

Prior investigations have already demonstrated the critical psychosocial importance of optical breast symmetry for patients [6–8]. Due to rapid progress in technology, a three-dimensional (3D) assessment has become feasible in assessing optical symmetry outcomes using digital anthropometry [9–11]. Nonetheless, most of the software provided to perform digital anthropometry is either custom-made and its use is often product-related or very time-consuming due to a lack of automatization.

This trial’s primary objective was to develop an independent piece of software, using standard 3D file formats, to assess breast symmetry by digital anthropometry automatically. The software was then used to compare outcomes in successful autologous and alloplastic BRS.

## Materials and Methods

### Study Design and Patients

The study was designed as a retrospective cohort study. All patients who had undergone BRS at our institution between January 2015 and January 2018 were included. Out of an initial cohort of 183 patients, 118 who had undergone either autologous or alloplastic BRS were selected. Patients with a history of flap-loss or implant-loss were excluded. One hundred and eighteen patients were invited to a specially arranged interview, and 27 agreed to be physically examined.

The alloplastic group (*n* = 15) included 12 patients who had received a successful immediate implant reconstruction and three patients who had received a successful two-stage reconstruction. Among these women, 13 had undergone bilateral and two unilateral BRS. Within this subgroup, ten patients had received a nipple-sparing mastectomy, while five patients received a skin-sparing subcutaneous mastectomy.

All implants were placed subpectorally using an acellular matrix in eight cases. In seven cases, the silicone gel breast implant was placed subpectorally without using a matrix.

The mean implant volume was 363 cc.

The autologous group (*n* = 12) included 12 patients who had undergone successful BRS with immediate Deep Inferior Epigastric Perforator Flap (DIEP-Flap). Within this group, 11 patients had undergone unilateral, while one had bilateral BRS. Within this subgroup, all patients had received a skin-sparing subcutaneous mastectomy.

Four patients had received consecutive contralateral mastopexy. The remaining patients did not receive an additional mastopexy.

The mean age was *M* = 48 years (SD =  ± 11), the mean height *M* = 167 cm (SD =  ± 6 cm), the mean weight *M* = 65.1 kg (SD =  ± 10.18 kg), and the mean BMI *M* = 23.1 (SD =  ± 4.1).

Before recruitment, the local ethics review board approval was obtained.

### Objective Symmetry Analysis

Two different anthropomorphic measurement methods were used to assess the outcomes' symmetry. Both were performed digitally. The results were then rated using a Symmetry Index (SI).

**Fig. 1:**
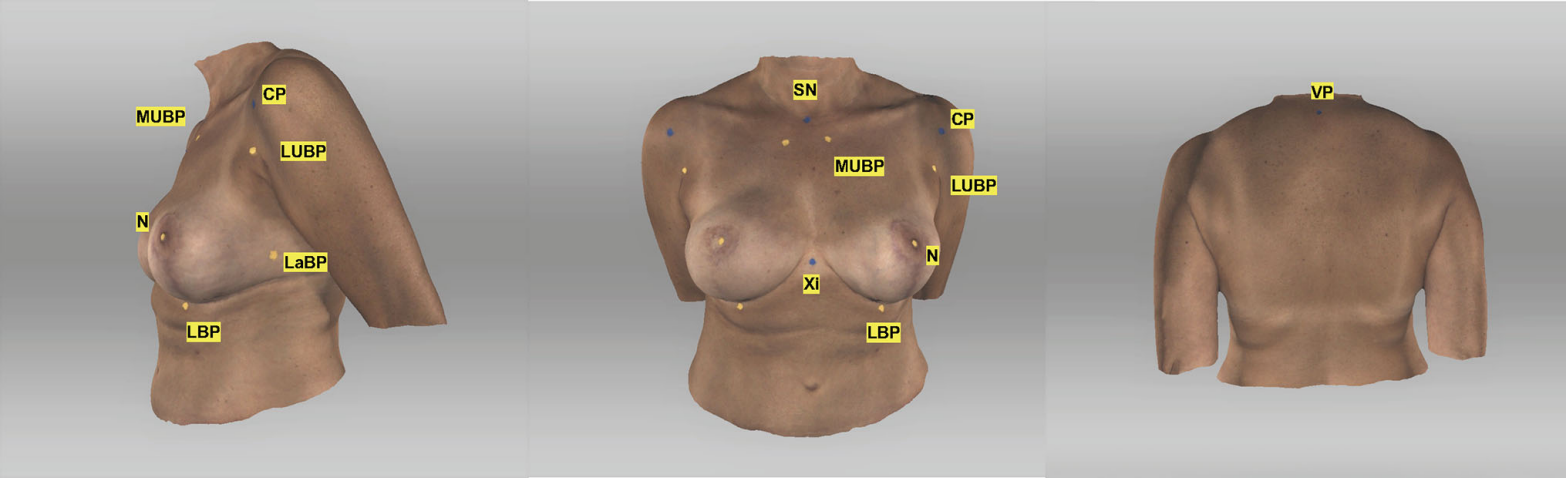
Landmarks. Appearance of a 54-year-old patient one year after DIEP-flap reconstruction R and mastopexy L; (1) sternal notch (SN), (2) medial upper breast pole (MUBP), (3) lateral upper breast pole (LUBP), (4) coracoid process (CP), (5) lateral breast pole (LaBP), (6) xiphoid (Xi), (7) lower breast pole (LBP), (8) nipple (N), and vertebra prominens (VP) (9); *Artec Studio 14* was used to create the illustration

Before examining the patient, nine landmarks as shown in Fig. 1 were defined to increase inter-rater reliability using yellow and blue-colored noncoded circular stickers.

The following landmarks were defined: (1) sternal notch (SN), (2) medial upper breast pole (MUBP), (3) lateral upper breast pole (LUBP), (4) coracoid process (CP), (5) lateral breast pole (LaBP), (6) xiphoid (Xi), (7) lower breast pole (LBP), (8) nipple (N), and (9) vertebra prominens (VP).

Landmarks (1), (4), (6), (8), (9) are static points. The remaining landmarks are defined in relation to (1), (4), (6), (8), (9). The MUBP is set 3 cm caudally and 2.5 cm laterally from the SN and the LUBP as the orthogonal intersection between a line through both MUBPs and the PC. The LBP is defined as the orthogonal intersection between the anterior axillary line and the nipple. The LBP marks the most caudal aspect of the inframammary fold (IMF).

If a patient had not received a Nipple-Areola-Complex (NAC) reconstruction, an ideal prospective NAC position was assumed by determining any missing NAC positions situated in the approximate middle of the breast gland vertically and slightly lateral to the midpoint horizontally [12] using yellow circular stickers.

Positions (1), (4), (7), (9) were labeled blue. The remaining landmarks were labeled yellow.

### 3D Assessment

The *Artec EVA* (Artec 3D, Luxembourg) was used for 3D assessment.

It is a handheld 3D scanner using structured light technique to create a virtual 3D model from the patient’s body surface information. According to the Artec Group (Artec 3D, Luxembourg), it measures 261.5 mm × 158.2 mm × 63.7 mm and weighs 850 g and reproduces the patient’s surface information with a reported accuracy of 0.5 mm [13].

It has shown high reproducibility in assessing 3D surfaces of the human body [14] and has been validated in a variety of studies for assessing breast morphology and shape [15–17].

Initially, the scanner creates a point cloud representing the patient’s body surface. By triangulation of the point cloud, a 3D mesh is generated, which is then used for further anthropomorphic measurements. For point cloud processing (such as noise reduction and outlier removal) and triangulation, *Artec Studio 14* (Artec 3D, Luxembourg) was used. Finally, the mesh was exported as a Wavefront OBJ (obj) file. Since the scanner used is able to capture color information as well, the resulting texture atlas is also saved.

The secondary editing was performed manually by cropping the virtual model above the elbow joints, at the thyroid cartilage and the abdomen through both anterior superior iliac spines.

### Semiautomatic Measurements

**Fig. 2:**
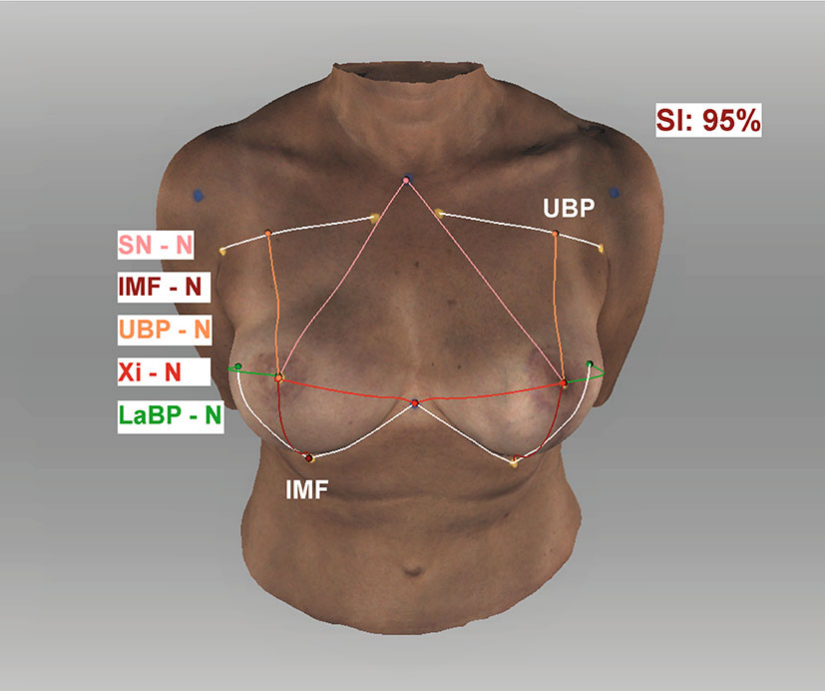
Semiautomatic measurements. Appearance of a 54-year-old patient one year after DIEP-flap reconstruction R and mastopexy L (1) sternal notch to nipple, (2) inframammary fold to nipple, (3) upper breast pole to nipple, (4) xiphoid to nipple, (5) lateral breast pole to nipple, (6) breast width (4 + 5), (7) inframammary fold length; Symmetry Index (SI); landmarks were detected manually; *Artec Studio 14* was used to create the illustration

**Fig. 3:**
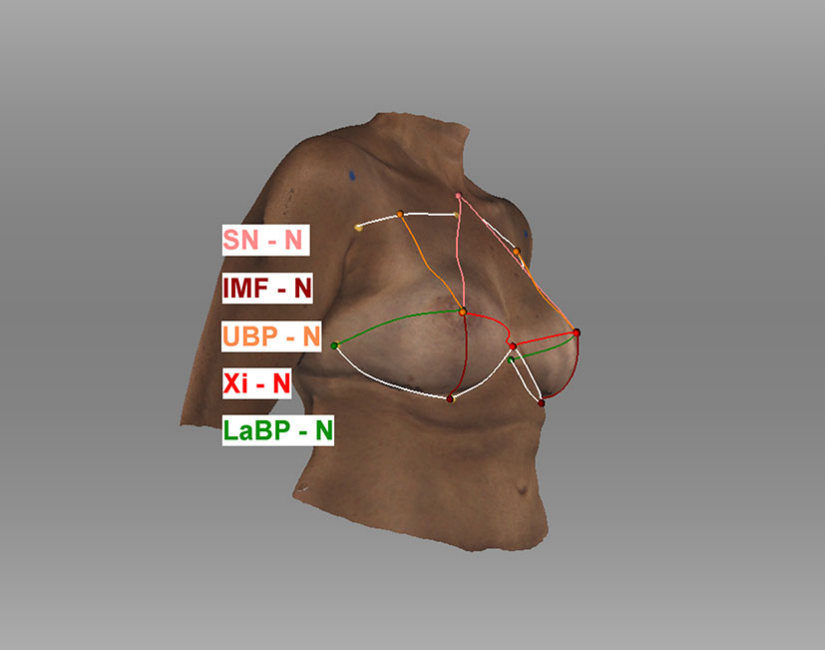
Semiautomatic measurements. Appearance of a 54-year-old patient one year after DIEP-flap reconstruction R and mastopexy L; lateral view; *Artec Studio 14* was used to create the illustration

**Fig. 4:**
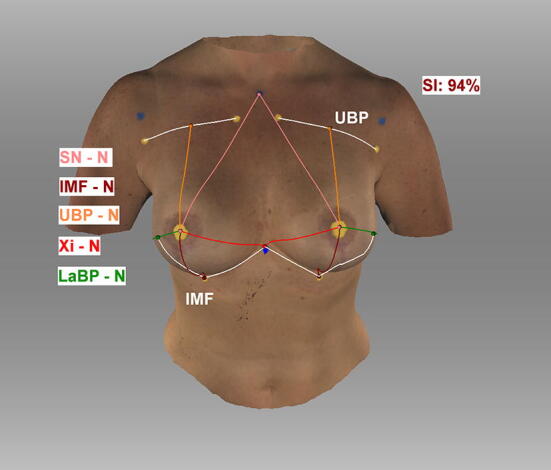
Semiautomatic measurements. Appearance of a 47-year-old patient three years after breast reconstruction using bilateral permanent silicone gel breast implant 350 cc R + L (1) sternal notch to nipple, (2) inframammary fold to nipple, (3) upper breast pole to nipple, (4) xiphoid to nipple, (5) lateral breast pole to nipple, (6) breast width (4 + 5), and (7) inframammary fold length; Symmetry Index (SI); landmarks were detected manually; *Artec Studio 14* was used to create the illustration

**Fig. 5:**
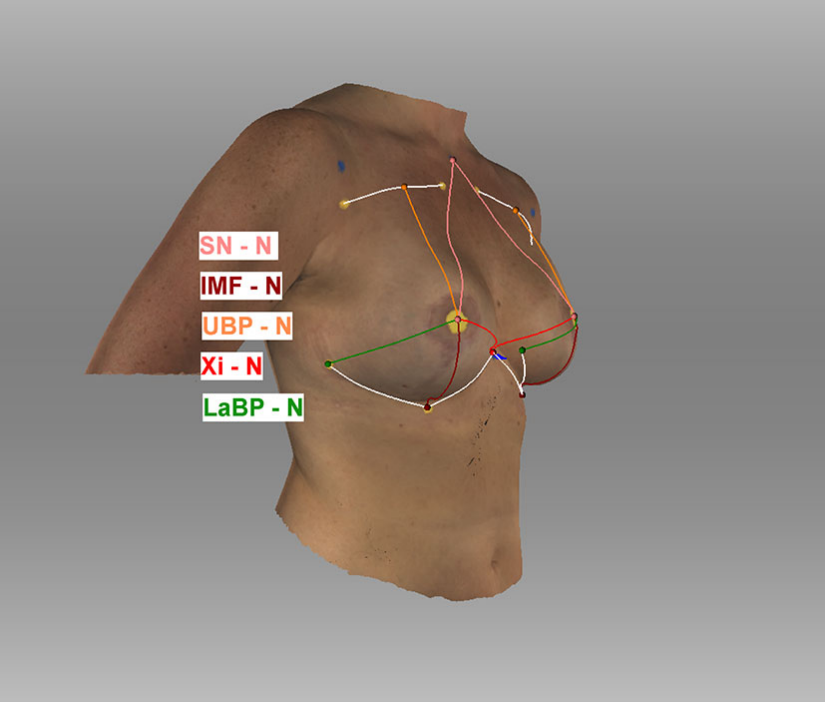
Semiautomatic measurements. Appearance of a 47-year-old patient three years after breast reconstruction using bilateral permanent silicone gel breast implant 350 cc R + L; lateral view; *Artec Studio 14* was used to create the illustration

The *Artec Studio measurement tool* (Artec 3D, Luxembourg) was used to perform semiautomatic anthropomorphic measurements on an input mesh. The geodesic distances between the landmarks were calculated by detecting the colored landmarks manually. Seven anthropomorphic measurements were taken: (1) sternal notch to nipple, (2) inframammary fold to nipple, (3) upper breast to nipple, (4) xiphoid to nipple, (5) lateral breast pole to nipple, (6) breast width, and (7) inframammary fold length (Figs. 2, 3, 4, 5).

### Automatic Measurements

**Fig. 6:**
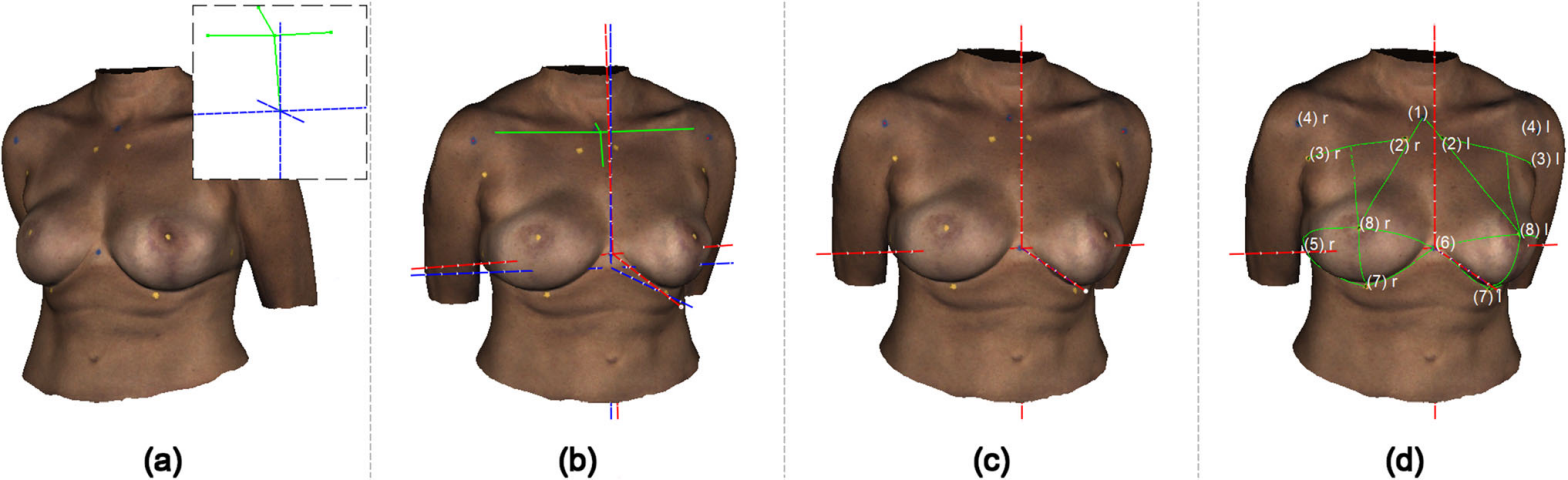
Authors proposed entirely automatized method for digital anthropomorphic measurements. **a** Textured input mesh with previously pasted stickers; top right corner: pre-defined template (green) used for global alignment. The template consists of landmarks 1, 4 (left and right), 6 and 9. **b** Input mesh after transformation into the global coordinate system (blue). The coordinate system (red) depicts the individual breast coordinate system. It is defined through the three anatomic main planes (sagittal, coronal, and transversal) and centered at xiphoid. **c** Input mesh after refining the initial global alignment, **d** labeled stickers and resulting anthropomorphic measurements

For automatic measurements, the standardized primary and secondary post-processing was performed as previously described. Landmarks were detected automatically on a textured input mesh using colored stickers and a sticker-detection algorithm (Fig. 6).

### Sticker-Detection Algorithm

The proposed algorithm consists of three stages:

First, a per-vertex color-thresholding is applied to select all vertices representing a sticker.

The selection is then split into independent sets of connected vertices, each one representing an individual sticker. Finally, midpoints are calculated for each sticker.

### Digital Anthropomorphic Measurements

The overall process is illustrated in Fig. 6.

Primarily, blue stickers were detected on the input mesh using the proposed sticker-detection algorithm. Subsequently, the stickers were used to transform the input mesh (Fig. 6a) into a global coordinate system (Fig. 6, blue) by registration with a pre-defined template (Fig. 6a, top right corner). The template consists of the anatomical landmarks (1), (4) (left and right), (6) and (9). The result of the global alignment is shown in Fig. 6b. The patient’s individual breast coordinate system (Fig. 6, red) defined through the three anatomical main planes (sagittal, coronal, and transversal) and centered at xiphoid is not well aligned with the global coordinate system. Therefore, the algorithm further refines the initial global alignment of the input mesh by aligning global and breast coordinate system (Fig. 6c). Both alignment steps are crucial for further processing since handheld 3D scanners create meshes of unknown orientation.

Step three included the detection of the yellow stickers using the previously described sticker-detection algorithm. Then, all detected stickers were labeled using a priori knowledge about the relative positions of the defined landmarks combined with a plane-sweep technique. Finally, measurements were taken using the labeled targets (Fig. 6d).

For the geodesic distance calculation, the algorithm proposed by Mitchell et al. [18] was used. After the calculation was performed, the algorithm outputted the measurements previously described as well as the SI.

### Symmetry Index

The SI is an instrument designed to evaluate outcomes among women undergoing different types of breast surgery. The idea behind the SI is to ensure robust breast symmetry analysis. The described standardized protocol for anthropomorphic measurements combines *N* anthropomorphic measurements into one indicative value, given *N* anthropomorphic measurements for the left and right mamma, where $${d}_{i}^{L}$$ ($${d}_{i}^{R})$$ denotes the $$i$$th measurement of the left (right) mamma ($$i\in \{1,2,\ldots ,n\}$$). The SI is then defined as the mean value of the ratios of *N* anthropomorphic measurements of both breasts:

$${\text{SI:}} = \frac{1}{N}\sum\limits_{{i = 1}}^{N} {\frac{{\min \left( {d_{i}^{L} ,d_{i}^{R} } \right)}}{{\max \left( {d_{i}^{L} ,d_{i}^{R} } \right)}}}$$

The SI is scored metrically with scores from 0 (worst) to 1 (best). It can be presented as a percentage by multiplying it by 100.

It is worth noting that prior symmetry indices have achieved statistically relevant results comparing seven parameters [19]. In this trial, seven anthropomorphic measurements were compared using the SI (*N* = 7).

For semiautomatic measurements, *Microsoft Excel* was used to calculate the SI. For automatic measurements, each obj file was processed using the developed software to determine its SI.

### Statistical Analysis

*IBM SPSS 25* was used to perform statistical analysis. A Wilcoxon–Mann–Whitney two-sample rank-sum test was used to compare the ages of the autologous and alloplastic groups. Median age for the alloplastic group was M = 39 (*n* = 15; SD =  ± 10.5) and *M* = 57.5 for the autologous group (*n* = 12; SD =  ± 8); the distributions in the two groups differed significantly (Mann–Whitney U = 22.0; *p* = 0.009).

The mean BMIs of the two groups were compared using Welch’s *t* test for equality of means. The mean BMI was *M* = 21.2 for the alloplastic group (*n* = 15; SD =  ± 2) and *M* = 25.5 for the autologous group (*n* = 12; SD =  ± 4.8); the distributions in the two groups differed significantly (Welch’s *t* test for independent samples; *p* = 0.01, two-tailed).

To compare the optical symmetry outcomes between the two techniques, SI scores of patients who had undergone successful autologous and alloplastic BRS were compared using the *t* test for equality of means.

The mean SI for semiautomatic measurements was *M* = 0.92 for the alloplastic group (*n* = 15; SD =  ± 0.02) and *M* = 0.91 for the autologous group (*n* = 12; SD =  ± 0.04) (Table 1); the distributions in the two groups did not differ significantly (*t* test for independent samples; *p* = 0.48, two-tailed) (Table 2).

**Table 1:** Group statistics; autologous and alloplastic groups compared by Symmetry Index

Operative technique	*N*	Mean	SD	SE mean
Symmetry Index (semiautomatic measurements)				
Autologous group	12	.91	.037	.011
Alloplastic group	15	.92	.022	.006
Symmetry Index (automatic measurements)				
Autologous group	12	.91	.033	.009
Alloplastic group	15	.93	.026	.007

The Symmetry Index was calculated for semiautomatic and automatic measurements; *IBM SPSS 25* was used for data analysis

**Table 2:** *T* test for independent samples; comparison of SI-means between autologous and alloplastic group

	*t* test for equality of means				
	Sig. (2-tailed)	Mean difference	SE difference	95% confidence interval of the difference	
				Lower	Upper
Symmetry Index (semiautomatic measurements)					
Equal variances assumed	.478	− .008	.012	25.000	.015
Symmetry Index (automatic measurements)					
Equal variances assumed	.082	− .021	.011	25.000	.003

Semiautomatic and automatic indices were tested separately; *IBM SPSS 25* was used for data analysis

The mean SI for automatic measurements was *M* = 0.93 for the alloplastic group (*n* = 15; SD =  ± 0.03) and *M* = 0.91 for the autologous group (*n* = 12; SD =  ± 0.03) (Table 1); the distributions in the two groups did not differ significantly (*t* test for independent samples; *p* = 0.08, two-tailed) (Table 2).

## Results

Successful autologous and alloplastic BRS outcomes were compared concerning optical symmetry. Statistical analysis revealed no difference in the optical symmetry of outcomes between the two groups (*t* test for independent samples; *p* = 0.48, two-tailed).

Patients in the alloplastic group were significantly younger than patients in the autologous group (Mann–Whitney *U* = 22.0; *p* = 0.009). Additionally, the BMI of patients in the alloplastic group was significantly lower (Welch’s *t* test for independent samples; *p* = 0.01) than those in the autologous group.

## Discussion

A limitation of this study is its small sample size. This was primarily due to the selection process. To obtain the comparable results for the two treatment groups, only similarly successful surgical outcomes were included. Consequently, patients with major complications, defined as flap- or implant-loss, were excluded from the investigation. This may on the one hand limit the reliability of the proposed symmetry assessment model.

On the other hand, the inclusion criteria guarantee the comparable results, as flap- or implant-loss inevitably affects an outcome’s symmetry. Besides, the response rate was relatively low since patients did not gain any financial benefit or any other profit from participating in this study. Furthermore, the additional examination may be an excessive psychosocial burden for a patient upon being confronted with her disease. However, the data were collected for patients and surgeons in the most suitable manner. Despite the small sample size, the present study may provide a foundation for utilizing three-dimensional analysis to compare distinct surgical approaches in BRS.

Other limitations involve the SI. Anthropomorphic measurements have been used widely in assessing the symmetry of outcomes [9, 10, 20, 21], and numerous formulae have been defined to evaluate such an outcome [8, 10]. Prior symmetry indices achieved the statistically relevant results comparing seven parameters [19]. Seven anthropomorphic measurements were used to create a reliable tool.

While the proposed data do provide a foundation for assessing the outcomes of BRS qualitatively, more studies need to be performed for further validation. In particular, further studies should investigate the impact of SI on different breast surgery procedures such as classic breast augmentation.

Another drawback of the proposed method may be the repositioning of the NAC. By assuming a prospective ideal position, the symmetry assessment may be biased.

Nonetheless, the NAC is positioned in accordance with the procedure commonly used when reconstructing a NAC [12]. This study includes successful BRS outcomes only. Therefore, the repositioning is an appropriate approximation used in this trial.

The authors’ findings on outcome symmetry are in concordance with previous investigations [11].

Evaluation of optical breast symmetry in patients is relevant in clinical practice. Nevertheless, various studies indicate that breast asymmetry occurs in most healthy women [22, 23]. Moreover, optical symmetry may not be as crucial to patients with a history of a potentially lethal disease.

Further studies should investigate patients’ desire for cosmetic factors when they are undergoing different breast surgery procedures. The SI must be classified as a tool to evaluate an outcome and must not be mistaken for an instrument to assess beauty or patients' satisfaction.

In addition, factors that could bias the results must be considered. For instance, the diverse periods between the reconstruction and the follow-up examination might affect a patient’s satisfaction as well as the outcome symmetry. It is worth noting that more women who received bilateral reconstruction underwent alloplastic BRS, while more women undergoing unilateral reconstruction underwent autologous procedures. Bilateral reconstruction might have an impact on the outcome symmetry as it simplifies intraoperative symmetry adjustments. Conversely, in women with unilateral reconstruction, a secondary contralateral correction was observed more often. This may explain why no significant differences in the outcome symmetry could be detected between the two therapeutic approaches. The trial sample size did not allow stratification for laterality.

In this investigation, patients in the alloplastic group were significantly younger (*p* = 0.009) than patients in the autologous group. Additionally, patients in the alloplastic group had a significantly lower BMI (*p* = 0.01). Age and BMI affect the breast morphology. This might have an effect on postoperative symmetry.

The study’s sample size did not allow stratification for age or BMI. However, larger studies may entail difficulties in measuring the SI in a subgroup of similar patients of the same age/BMI, as these factors determine the therapeutic options.

Microvascular autologous reconstruction requires a sufficient donor site. Patients with a smaller amount of skin and subcutaneous fat (average to thin body habitus) are often advised to undergo alloplastic BRS. Previous studies show that younger women are more likely to undergo alloplastic BRS [24, 25]. Consequently, stratification for age and BMI is problematic to realize.

Unlike in previous approaches to sufficient breast morphology and symmetry analysis [8, 9, 26–28], a fully automatized method based on portable 3D scanning devices is introduced here. In contrast to the scan systems used in previous studies, handheld devices created a mesh of unknown orientation for the present study. The novel software allows clinicians to make use of meshes generated by handheld devices, decreasing errors by fully automatizing the measurements.

Moreover, the proposed method is aimed at contributing to the advancement of postoperative outcome evaluation in successful BRS by introducing a reproducible method to assess breast symmetry. Additionally, the SI can be applied also to semiautomatic and analogous measurements, guaranteeing its universal use.

Nonetheless, primary landmark detection still depends on the examiner's accuracy. Accordingly, the improvement in automatic anatomical landmark detection is required. Finally, this work aims to draw attention to the lack of postoperative evaluation in BRS. By developing a new method to quantify symmetry objectively, this trial aspires to contribute to the advancement of postoperative outcome evaluation in BRS.

## Conclusion

The proposed data does provide a foundation for beginning to qualitatively assess the outcomes in BRS using automatized digital anthropometry. In the present trial, no difference in the outcomes’ optical symmetry was detected between autologous and alloplastic BRS.

Breast reconstruction surgery is more than an operative technique and therefore requires a plastic surgeon's ability to provide information concerning the procedure. It is with this belief that we developed an innovative tool to assess outcomes objectively.

## References

[1] Bray F, Ferlay J, Soerjomataram I, Siegel RL, Torre LA, Jemal A (2018). Global cancer statistics 2018: GLOBOCAN estimates of incidence and mortality worldwide for 36 cancers in 185 countries. CA Cancer J Clin.

[2] Barnes B, Robert-Koch-Institut (2016) Bericht zum Krebsgeschehen in Deutschland 2016. Robert Koch-Institut, Berlin

[3] Gerber B, Marx M, Untch M, Faridi A (2015). Breast reconstruction following cancer treatment. Dtsch Aerzteblatt Online.

[4] Fallbjörk U, Karlsson S, Salander P, Rasmussen BH (2010). Differences between women who have and have not undergone breast reconstruction after mastectomy due to breast cancer. Acta Oncol.

[5] Nahabedian MY, Galdino G (2003). Symmetrical breast reconstruction: is there a role for three-dimensional digital photography?. Plast Reconstr Surg.

[6] Chan W, Mathur B, Slade-Sharman D, Ramakrishnan V (2011). Developmental breast asymmetry: developmental breast asymmetry. Breast J.

[7] Neto MS, Abla LEF, Lemos AL, Garcia ÉB, Enout MJR, Cabral NC, Ferreira LM (2012). The impact of surgical treatment on the self-esteem of patients with breast hypertrophy, hypomastia, or breast asymmetry. Aesthetic Plast Surg.

[8] Pei J, Fan J, Ashdown SP (2019). A novel method to assess breast shape and breast asymmetry. J Text Inst.

[9] Liu C, Luan J, Mu L, Ji K (2010). The role of three-dimensional scanning technique in evaluation of breast asymmetry in breast augmentation: a 100-case study. Plast Reconstr Surg.

[10] Yeslev M, Braun SA, Patrick Maxwell G (2016). Asymmetry of inframammary folds in patients undergoing augmentation mammaplasty. Aesthet Surg J.

[11] Cohen O, Small K, Lee C, Petruolo O, Karp N, Choi M (2016). Is unilateral implant or autologous breast reconstruction better in obtaining breast symmetry?. Breast J.

[12] Lewin R, Amoroso M, Plate N, Trogen C, Selvaggi G (2016). The aesthetically ideal position of the nipple-areola complex on the breast. Aesthetic Plast Surg.

[13] Modabber A, Peters F, Kniha K, Goloborodko E, Ghassemi A, Lethaus B, Hölzle F, Möhlhenrich SC (2016). Evaluation of the accuracy of a mobile and a stationary system for three-dimensional facial scanning. J Cranio Maxillofac Surg.

[14] Verhulst A, Hol M, Vreeken R, Becking A, Ulrich D, Maal T (2018). Three-dimensional imaging of the face: a comparison between three different imaging modalities. Aesthet Surg J.

[15] Chen K, Feng C-J, Ma H, Hsiao F-Y, Tseng L-M, Tsai Y-F, Lin Y-S, Huang L-Y, Yu W-C, Perng C-K (2019). Preoperative breast volume evaluation of one-stage immediate breast reconstruction using three-dimensional surface imaging and a printed mold. J Chin Med Assoc.

[16] Koban K, Schenck T, Metz P, Volkmer E, Haertnagl F, Titze V, Giunta R (2016). Auf dem Weg zur objektiven evaluation von form, volumen und symmetrie in der plastischen chirurgie mittels intraoperativer 3D scans. Handchir Mikrochir Plast Chir.

[17] Oranges CM, Madduri S, Brantner P, Msallem B, Giordano S, Benitez B, Kalbermatten DF, Schaefer DJ, Thieringer FM (2019). Three-dimensional assessment of the breast: validation of a novel, simple and inexpensive scanning process. In Vivo.

[18] Mitchell JSB, Mount DM, Papadimitriou CH (1987). The discrete geodesic problem. SIAM J Comput.

[19] Swobodnik Alexandra (2012) Standardisierung einer objektiven 3-D Evaluationsmethode und Entwicklung eines Symmetrieindex zur Beurteilung von Brustasymmetrien in der Plastischen, Rekonstruktiven und Aesthetischen Chirurgie. TUM

[20] Malata CM, Boot JC, Bradbury ET, Ramli ARB, Sharpe DT (1994). Congenital breast asymmetry: subjective and objective assessment. Br J Plast Surg.

[21] Stark B, Olivari N (1991). Breast asymmetry: an objective analysis of postoperative results. Eur J Plast Surg.

[22] Rohrich RJ, Hartley W, Brown S (2003). Incidence of breast and chest wall asymmetry in breast augmentation: a retrospective analysis of 100 patients. Plast Reconstr Surg.

[23] Gabriel A, Fritzsche S, Creasman C, Baqai W, Mordaunt D, Maxwell GP (2011). Incidence of breast and chest wall asymmetries: 4D photography. Aesthet Surg J.

[24] Manrique OJ, Charafeddine A, Abu-Ghname A, Banuelos J, Jacobson SR, Martinez-Jorge J, Nguyen M-D, Harless C, Tran NV, Sharaf B, Jakub JW, Hieken TJ, Degnim AC, Boughey JC (2019). Two-staged implant-based breast reconstruction: a long-term outcome study in a young population. Medicina (Mex).

[25] Doherty C, Pearce S, Baxter N, Knowles S, Ross D, McClure JA, Brackstone M (2019). Trends in immediate breast reconstruction and radiation after mastectomy: a population study. Breast J.

[26] Kovacs L, Eder M, Hollweck R, Zimmermann A, Settles M, Schneider A, Udosic K, Schwenzer-Zimmerer K, Papadopulos NA, Biemer E (2006). New aspects of breast volume measurement using 3-dimensional surface imaging. Ann Plast Surg.

[27] Kovacs L, Yassouridis A, Zimmermann A, Brockmann G, Wöhnl A, Blaschke M, Eder M, Schwenzer-Zimmerer K, Rosenberg R, Papadopulos NA, Biemer E (2006). Optimization of 3-dimensional imaging of the breast region with 3-dimensional laser scanners. Ann Plast Surg.

[28] Eder M, Klöppel M, Müller D, Papadopulos NA, Machens H-G, Kovacs L (2013). 3-D analysis of breast morphology changes after inverted T-scar and vertical-scar reduction mammaplasty over 12 months. J Plast Reconstr Aesthet Surg.

